# Experience in Rehabilitation Treatment for Patients Repeatedly Admitted to Intensive Care Units After Dual Graft Living-Donor Liver Transplantation: A Case Report

**DOI:** 10.7759/cureus.77099

**Published:** 2025-01-07

**Authors:** Makoto Asaeda, Shunsuke Taito, Akihiro Matsumoto, Yuki Nakashima, Koki Fukuhara, Kenichi Fudeyasu, Tomoya Hirai, Yukio Mikami

**Affiliations:** 1 Department of Rehabilitation Medicine, Hiroshima University Hospital, Hiroshima, JPN; 2 Division of Rehabilitation, Department of Clinical Practice and Support, Hiroshima University Hospital, Hiroshima, JPN; 3 Department of Rehabilitation, Osakafu Saiseikai Ibaraki Hospital, Osaka, JPN

**Keywords:** bile peritonitis, icu-acquired weakness, living-donor liver transplantation (ldlt), rehabilitation program, septic shock (ss)

## Abstract

Living-donor liver transplantation (LDLT) has become a common procedure in Japan, allowing patients to maintain their activities of daily living (ADL) after surgery. However, complications, such as bile leakage and septic shock, often occur, necessitating intensive rehabilitation. This case report details the rehabilitation of a woman in her 60s who experienced peritonitis and recurrent septic shock after LDLT, with the goal of providing insights for future rehabilitation protocols.

The patient had a preoperative Child-Pugh class C and Model for End-Stage Liver Disease score of 21. Preoperative rehabilitation included gait and muscle-strengthening exercises. After a 15-hour dual-graft LDLT, she experienced multiple postoperative complications, including bile leakage on day X+22, requiring resuturing and multiple intensive care unit (ICU) admissions. Rehabilitation was adjusted to suit her ICU condition, with exercises, such as range-of-motion and assisted walking, based on her ICU mobility score. After her final ICU discharge on day X+55, she continued rehabilitation in a general ward, progressing from wheelchair transfer to walking with a walker, which resulted in Barthel index improvement from 0 to 55 on day X+133. Despite improved ADL, she experienced muscle atrophy, particularly in the psoas muscle, due to ICU-acquired weakness and prolonged inactivity.

This case highlights the importance of individualized rehabilitation approaches in patients with LDLT and associated complications. Given the lack of specific post-LDLT guidelines, particularly for patients with ICU-AW, this report highlights the need for objective indicators, such as heart rate control and muscle strength assessments, to guide rehabilitation. Traditional methods have proven effective in improving ADL; however, further strategies are needed to address muscle mass recovery. This case suggests that a tailored approach can improve patient outcomes and provide valuable insights into the development of LDLT-specific rehabilitation guidelines.

## Introduction

As of 2017, 8,795 living donor liver transplants have been performed in Japan, and this procedure has become widely recognized [1]. After living-donor liver transplantation (LDLT), patients can maintain a certain level of activities of daily living (ADL), as indicated by the Barthel index, which decreases by only 5 points on average after a hospital stay of 54.2 days [2]. Therefore, with appropriate postoperative management, patients often experience good rehabilitation outcomes. However, complications frequently occur after LDLT, with bile leakage and gastric content stasis being the most common, which occur at a rate of 12% [3]. In particular, a preoperative decline in physical function is thought to be associated with complications, and skeletal muscle atrophy, as observed on computed tomography (CT), has been reported to be associated with postoperative infections [4]. As many patients requiring LDLT already have a decline in their performance status before surgery [5,6], it is expected that facilities performing this procedure will frequently encounter patients requiring rehabilitation treatment for postoperative complications. However, there have been no reports on rehabilitation treatment, body structure and function, and ability according to the International Classification of Functioning, Disability, and Health, which is a framework for describing and organizing information on function and disability in patients who have developed postoperative complications requiring intensive care management after LDLT.

In this study, we report the rehabilitation treatment of a patient who developed peritonitis due to bile leakage and multiple episodes of septic shock after LDLT with the aim of establishing future rehabilitation treatments for such patients.

## Case presentation

Patient information

This case report is in accordance with the CAse REports guidelines [7,8]. The patient is a woman in her 60s with a height of 156.3 cm and a weight of 64.8 kg. She experienced loss of appetite and fatigue five months before surgery. Three months before the surgery, she had hematuria and jaundice and visited a doctor, where CT revealed a tumorous lesion in the right lobe of the liver. A blood test indicated prolonged prothrombin time. She was referred to our hospital’s gastroenterology department 63 days before the surgery and was admitted for further examination 57 days before the surgery. Although various tests, including CT, gastrointestinal endoscopy, and liver biopsy, were performed, no obvious tumorous lesions were found (liver biopsy showed no Epstein-Barr encoding region-positive cells in the hepatic tissue on immunohistochemistry). She had a fever, which started 32 days before the surgery. Her blood pressure decreased the next day, resulting in sepsis and admission to the high-care unit (HCU). A consultation with the rehabilitation department was performed 21 days before the surgery, and physical and occupational therapies were initiated. At the time of intervention, her Child-Pugh classification was class C (10), and the model for end-stage liver disease score was 21. Her medical history included premature ventricular contractions (PVCs) and hypertension, with no notable exercise or rehabilitation history. Echocardiography revealed an ejection fraction of 69%. Before surgery, the patient underwent treatment for infection and fever, including medication and paracentesis for ascites. No steroids or immunosuppressants were used preoperatively.

Timeline

Liver Transportation

On day X, living-related liver transplantation with dual grafts from two donors was performed (both the left and caudate lobes). Surgery was performed under general anesthesia, and a large amount of bloody ascites was observed during the operation. The duration of the operation was 15 hours and 47 minutes, with a blood loss of 4,193 g and a transfusion of 14 units of red blood cells and 16 units of plasma products.

After Liver Transportation

Postoperative courses are shown in Table 1. Postoperatively, the patient was extubated as planned, and on day X+5, rehabilitation was initiated by sitting at the edge of the bed, showing a smooth postoperative course. On day X+17, the patient was transferred to the general ward. However, on day X+22, the patient experienced worsening abdominal pain and was diagnosed with diffuse peritonitis due to an anastomotic leak at the choledochojejunostomy site, necessitating resuturing surgery. Postoperatively, the patient was readmitted to a surgical intensive care unit (ICU) and required mechanical ventilation. On day X+30, the patient was transferred back to the general ward. On day X+41, the patient was readmitted to the ICU for restenting of the bile duct for the right lobe graft. After returning to the general ward once on day X+51, the patient experienced septic shock and was admitted to the HCU for a fourth time for comprehensive management. After returning to the general ward on day X+55, her condition did not deteriorate, and the patient gradually began rehabilitation and mobilization. On day X+133, the patient was transferred to another general hospital to continue rehabilitation treatment. Postoperatively, steroid and immunosuppressant therapy was administered, with dosages gradually tapered. Additionally, antibiotics were prescribed based on susceptibility.

**Table 1:** Timeline after liver transportation hANP: human atrial natriuretic peptide; CMV: cytomegalovirus; HCU: high care unit; ICU: intensive care unit; SICU: surgical intensive care unit; CT: computed tomography; sBP: systolic blood pressure

Day	Event
X+1	Tracheal extubation and initiation of hANP
X+3	Removal of gastric tube
X+4	Removal of blood access and insertion of peripherally inserted central venous catheter
X+5	Sitting on the edge of the bed
X+7	Standing
X+8	Black stool, anemia, and hypotension; confirmation of extravasation on CT; initiation of ganciclovir for CMV viremia; conservative treatment for bleeding
X+13	Transfer from SICU to HCU
X+14	Stepping in place
X+16	Walking
X+15	Presence of pleural effusion; drainage performed
X+17	Transfer to general ward
X+22	Worsening abdominal pain, tachypnea; large amount of pleural effusion and ascites on CT; resuturing surgery for bile-jejunum anastomotic insufficiency due to diffuse peritonitis; admission to SCIU; management with mechanical ventilation
X+24	Extubation, resumption of sitting on the edge of the bed, and standing
X+27	Transfer from SICU to HCU
X+30	Transfer to general ward
X+41	Reinsertion of drainage and biliary stent tube (right graft), transfer to ICU
X+43	Transfer to general ward
X+44	Staring meal
X+51	Shivering and fever >38°C, hypotension (sBP = 60 mmHg); admission to HCU due to septic shock
X+54	Sitting on a wheelchair
X+55	Transfer to general ward
X+70	Initiation of wheelchair sitting during meals
X+91	Rehabilitation conference (keeping mobilization by all staffs)
X+93	Ward conference: rehabilitation before lunch, followed by meal intake
X+98	Walker-assisted walking training
X+114	Endurance training
X+119	Muscle strength exercise by machine
X+133	Transfer to a general hospital for continuation of rehabilitation treatment

Therapeutic Intervention

Physical preoperative occupational therapy: Before LDLT, physical therapy included gait training and muscle strengthening exercises. On day X-17, the patient could walk 30 m using a walker and became independent while walking with a walker in the ward. From day X-14, the patient could walk 30 m without upper limb support. Occupational therapy included ADL and gait training. From day X-14, the patient started activity training in a wheelchair and was able to perform continuous seated activities for 40 minutes.

Rehabilitation treatment while admitted into ICU or HCU: Rehabilitation treatments administered during the stay in the HCU and ICU are shown in Table 2. The rehabilitation program was conducted according to the activity restrictions of the respective departments. Until the fourth postoperative day, the patient was on bed rest, and on the fifth day, activity levels increased; rehabilitation was conducted based on the ICU mobility score (IMS). The IMS is a quick and simple method for measuring the mobility of critically ill patients at the bedside. It is a reliable evaluation tool for assessing the mobility of patients in the ICU, rated on an 11-point scale ranging between 0 and 10 [7,8]. In this case, rehabilitation in the HCU and ICU aimed at early mobilization at the highest functional level, starting with sitting at the edge of the bed. Until postoperative day 17, the patient’s activity levels gradually increased according to the postoperative protocol. The same rehabilitation was performed during bile peritonitis and septic shock postoperatively and was extended to standing exercises. She was able to expel sputum independently and did not experience oxygenation decline or complications such as pneumonia; therefore, no specific respiratory training was conducted.

**Table 2:** Rehabilitation treatment while admitted to ICU or HCU HCU: high care unit; ICU: intensive care unit; SICU: surgical intensive care unit

Room	Admission reason	Admission period (day)	Rehabilitation treatment
HCU	Septic shock	2	Range of motion exercise, mobilization, walking (100 m)
SICU→HCU	Living-donor liver transplantation	17	Range of motion exercise and mobilization to standing and walking (5 m)
SICU→HCU	Bile peritonitis due to incomplete suturing of the biliary-enteric anastomosis	8	Range of motion exercise and mobilization to standing
ICU	Dysfunction of drainage, replacement of drain, and reinsertion of biliary stent tube	2	Not performed
HCU	Septic shock	4	Mobilization to standing

Rehabilitation treatment after fifth HCU admission: After treatment in the HCU for the fifth time, rehabilitation was resumed in the general ward. Rehabilitation treatment was conducted five days a week for at least 40 minutes per session, with sessions lasting up to 80 minutes. On weekends, the patient performed 20 minutes of training in her room each day. From discharge from the HCU after the fifth admission to another hospital, 76 days of rehabilitation treatment and 70 sessions were provided. Regarding exercise load, Shindo et al. recommended that for rehabilitation of patients with critical illness polyneuropathy after LDLT, the heart rate should be <75%-80% of the maximum heart rate: using the Karvonen formula: Target heart rate = ((220 - age) - Resting heart rate (100 bpm)) × 0.8 + Resting heart rate (100 bpm) ≒ 130 bpm [9], SpO_2_ should not decrease, the Borg scale should be <12-13, and there should be no signs of overexertion, such as muscle weakness [10]. In this case, the resting heart rate fluctuated around 100-120 bpm, and an exercise load of up to 130 bpm was allowed. Strength, basic movements, ADL living, and endurance training were performed.

**Figure 1:**
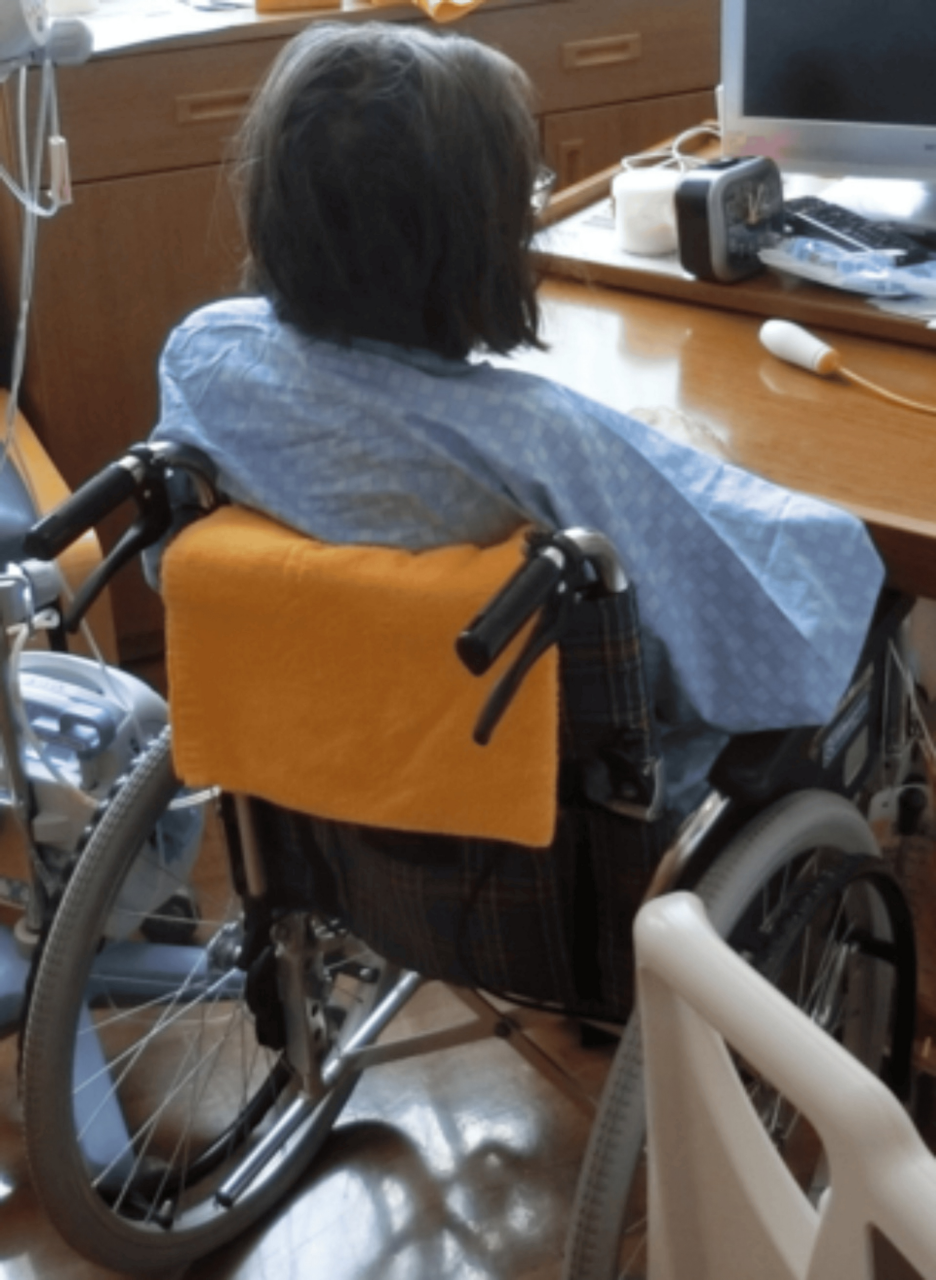
Transfer to a wheelchair for meal intake

**Figure 2:**
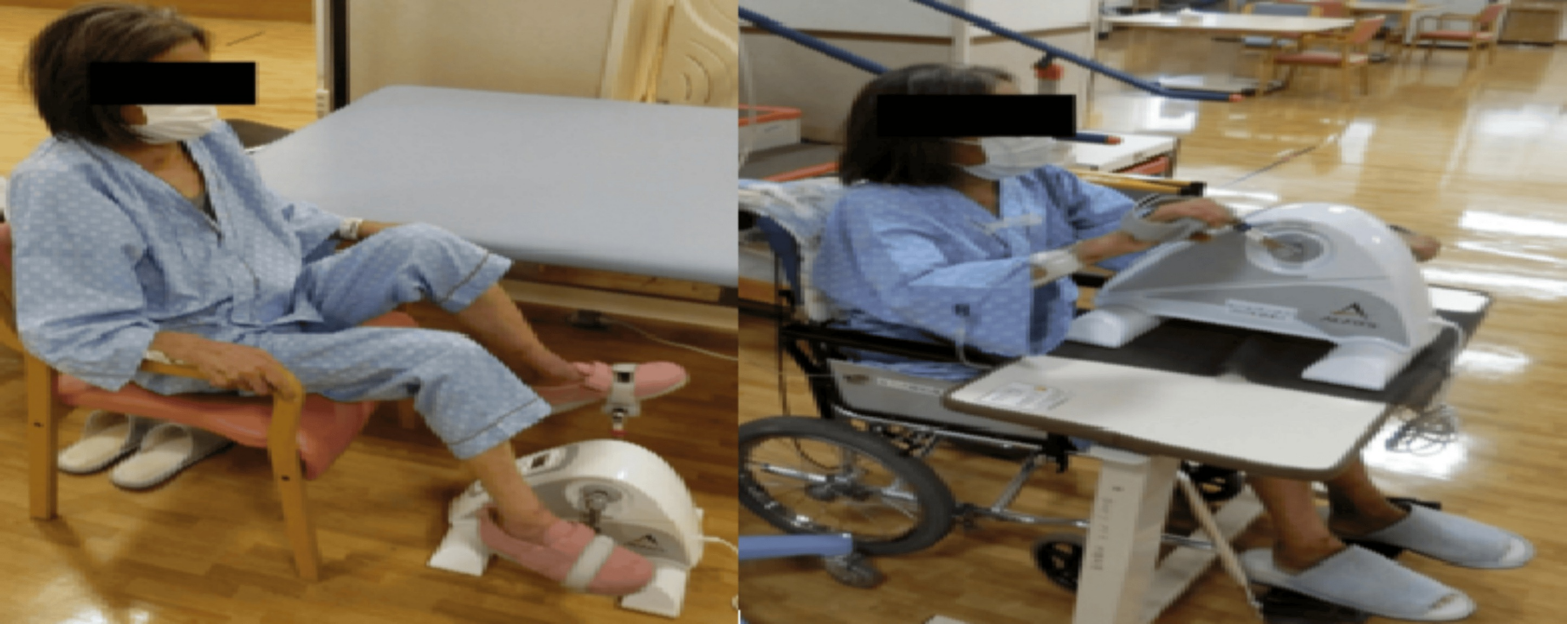
Aerobic exercise. Left: lower limb ergometer. Right: upper limb ergometer

At the start of rehabilitation, she could not roll in bed or stand by herself; therefore, we planned the rehabilitation to recover her ADL. We began by assisting with the rolling, sitting up, and maintaining a sitting position at the edge of the bed. During bed-edge sitting, PVC occurred, and her heart rate exceeded 130 bpm. Therefore, we used a reclining wheelchair. Gradually, we extended her sitting time in the reclining wheelchair. From day X+70, since her heart rate remained <130 bpm, we instructed the nursing staff to transfer her to the wheelchair during lunch to ensure adequate sitting time and promote eating (Figure 1). After becoming able to transfer to the wheelchair, the rehabilitation treatment was moved to the rehabilitation room, where seated muscle strength exercises and standing exercises were conducted. Gait training began on day X+98, and a walker was used to prevent falls. With continuous 5-m walking, her heart rate increased to 130 bpm, so the plan was to gradually increase the walking distance. By day X+114, she was able to walk continuously for 30 m with a walker, and aerobic exercises with hand or leg ergometers were initiated (Figure 2). The exercise load began at the minimum level, with exercise interrupted if PVCs occurred or if her heart rate reached 130 bpm under ECG monitoring. Accordingly, the Borg scale increased to approximately 13 after exercise.

Follow-Up and Outcomes

**Figure 3:**
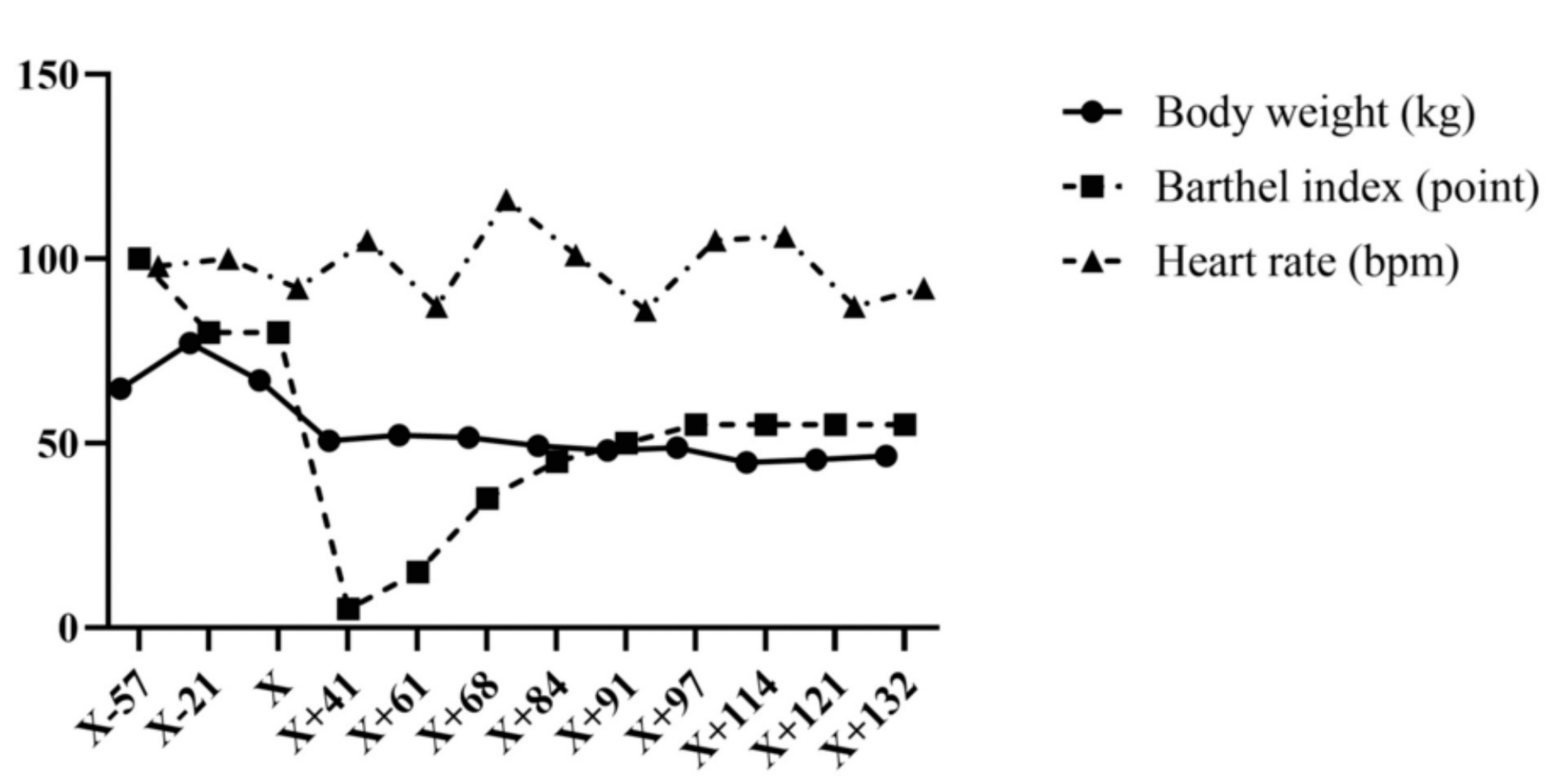
Body weight and the Barthel index

Changes in weight and Barthel index from the start of preoperative physical therapy to transfer to another hospital are shown in Figure 3. The Barthel index was initially at full score, but the ADL level gradually decreased before the surgery. Following the LDLT, owing to multiple admissions to the HCU, the Barthel index was 0. After the fifth discharge from the HCU, the Barthel index increased to 55 as bowel control improved, the urinary catheter was removed, independence in eating and grooming activities was achieved, and assistance with wheelchair transfer decreased. Regarding weight, there were significant ascites preoperatively, resulting in a high weight. However, postoperatively, the weight was lower than that before the surgery, and it gradually decreased. The resting heart rate showed a temporary increase without a gradual reduction.

The Medical Research Council (MRC) scores during treatment are shown in Table 3. Preoperatively, the patient had mild muscle weakness in both the upper and lower limbs. On day X+17, the first discharge from the HCU after transplantation, further muscle weakness was observed in the lower limbs. However, the patient was able to stand and walk a few meters. By day X+55, after multiple admissions and discharges from the HCU, there was further muscle weakness in both the upper and lower limbs, and some parts of the limbs had difficulty with antigravity movement. With continuing rehabilitation treatment, by day X+132, muscle strength improvement was observed, but it did not recover to the level before surgery or immediately after the first HCU discharge.

**Table 3:** MRC score (left side/right side) MRC: medical research score

Motion	X-21	X+17	X+55	X+132
Shoulder abduction	4/4	4/4	2/2	3/3
Elbow flexion	4/4	4/4	3/3	3/3
Wrist dorsiflexion	4/4	4/4	3/3	3/3
Hip flexion	4/4	3/3	2/3	3/3
Knee extension	4/4	3/3	3/2	3/3
Ankle dorsiflexion	4/4	3/3	2/2	3/3
Total score	48	42	30	36

The results of physical function and skeletal muscle mass (bioimpedance method, CT) are shown in Tables 4, 5. Grip strength and knee extension strength were lower than those preoperatively, and physical performance indices, such as the five-time chair stand and short physical performance battery, could not be performed postoperatively because of decreased physical function. Regarding skeletal muscle mass, the bioimpedance method (InBody S10, Japan) showed higher values preoperatively, owing to the influence of preoperative ascites, whereas lower muscle mass was observed postoperatively. For the cross-sectional area of the psoas major muscle on CT, the decrease from just before LDLT to the first discharge from the HCU after transplantation was greater than that from the first discharge to the final discharge from the HCU. The five-times sit-to-stand test was deemed “not feasible” because the patient had difficulty standing up using the handrail after surgery. Regarding the six-minute walk test, the patient was able to walk continuously with occasional rest before the surgery. However, after surgery, the patient could only walk 30 m under supervision with a walker, leading to a determination that the test could not be conducted. Furthermore, walking speed did not improve after surgery; instead, it was slower at the time of transfer to another hospital than immediately after surgery. Muscle mass recovery did not occur until the patient was transferred to another hospital.

**Table 4:** Pre- and postoperative physical function and body composition SPPB: short physical performance battery; SMI: skeletal muscle mass index

Outcome	Preoperative (X-12)	Transfer (X+132)
Grip strength (kg)	20.9	12.0
Knee extension strength (kgf/body weight)	0.15	0.11
Five-times sit-to-stand test (seconds)	15.94	Not feasible
SPPB score	7	0
Six-minute walk test (m)	60	Not feasible (30)
SMI	9.05	4.16

**Table 5:** Volume of psoas muscle by CT CT: computed tomography

Outcome	X-4	X+16	X+52	X+89	X+127
Area	10.41	8.93	4.88	5.55	5.20
Index	4.26	3.66	2.00	2.27	2.13

## Discussion

This case involved a patient who required long-term intensive care management due to the postoperative complications of septic shock and bile peritonitis caused by incomplete suturing of the biliary-enteric anastomosis after LDLT. In the guidelines for acute-on-chronic liver failure after liver transplantation, it is recommended to perform exercise, sit up, and other functional training on the bed when there is no consciousness disorder but there is a decrease in limb muscle strength [11]. However, there are no specific guidelines for rehabilitation therapy after LDLT, and the treatment content is mostly determined based on the overall perioperative condition. This case study is the first to report the practice of rehabilitation therapy based on specific exercise indices and outcomes related to body structure, function, and activity.

In our hospital, we implemented bed rest for the first two days after the surgery [12] and prevented contracture by sitting up and performing a joint range of motion exercises based on a previous randomized controlled trial showing its effectiveness in achieving early mobilization to sitting [13]. Gradual mobilization was started on the third postoperative day. In this case, we initiated gradual mobilization considering the overall condition of the patient. However, on postoperative day 22, the patient developed diffuse peritonitis due to bile peritonitis caused by incomplete suturing of the biliary-enteric anastomosis, necessitating readmission into the HCU due to septic shock. The incidence of bile leakage after LDLT is 8.2%, with most cases occurring within one month postoperatively [14]. Therefore, the occurrence of peritonitis due to bile leakage after LDLT is relatively predictable. However, in this case, considering the decrease in the MRC score and cross-sectional area of the psoas major muscle on CT after prolonged intensive care, it is also possible that the patient experienced ICU-acquired weakness. The probability of developing ICU-AW after LDLT is 2.5% [15], and ICU-AW is considered relatively rare in conjunction with the aforementioned postoperative complications.

As for the rehabilitation treatment in this case, based on previous studies [10], we implemented strengthening exercises, basic movement training, activities of daily living training, and endurance training, ensuring that exercise loads remained below 130 bpm. It is recommended to maintain the intensity of aerobic exercise at two to four metabolic equivalents (METs) when the intensity is <4 METs postoperatively [11]. Intense exercise equivalent to 80% VO_2_ max significantly increases coagulation ability immediately after exercise and 30 minutes after exercise [16]. However, moderate exercise (equivalent to 55% VO_2_ max) did not cause such changes [16], which is considered an important aspect of the rehabilitation treatment in this case. The introduction of the Enhanced Recovery After Surgery protocol can shorten the ICU admission period compared to conventional interventions [17], but it cannot deny the possibility of excessive whole-body energy demand, inflammation due to decreased organ blood flow, and delayed wound healing caused by exercise overload. Additionally, this case may have been complicated by ICU-AW, and relatively light exercise therapies, such as cycle ergometry and resistance training, have been reported to be effective for ICU-AW [18]. The patient gradually implemented exercises from daily life activities, aiming for 80% of the predicted maximum heart rate of 130 bpm, and her Barthel index and MRC score improved. However, the lack of a sufficient load on the muscle tissue may have prevented an increase in muscle mass and body weight. The cutoff value for the psoas major muscle index (PMI) by CT is 425.72 mm^2^/m for men and 364.14 mm^2^/m for women [19], and sarcopenia can be detected in 36% of patients with cirrhosis using PMI [20]. Furthermore, patients with low skeletal muscle mass before liver transplantation have a lower survival rate during the follow-up period, and skeletal muscle mass is a prognostic factor after liver transplantation [21]. Therefore, it is necessary to improve long-term clinical outcomes in this case by gradually increasing the exercise load in a state where the overall condition is calm, and the resting heart rate is reduced.

The strengths of the rehabilitation treatment, in this case, include reporting rehabilitation medicine that clarifies objective indicators and outcomes for the decrease in physical function due to postoperative complications after LDLT, observing a gradual improvement in ADL despite multiple admissions to the HCU, and showing a low correlation between ADL improvement and muscle mass increase. However, the weaknesses include not being able to clearly determine whether the decrease in postoperative physical function was due to disuse or ICU-AW, not being able to clarify the recovery process to the pre-disease state, and not implementing electrical stimulation therapy for ICU-AW [22], which is considered effective. Therefore, only classical rehabilitation therapy was implemented. This case study is the first to report on the practice of rehabilitation therapy based on specific exercise indicators and outcomes related to body structure, function, and activity. Although this study focused on a patient who underwent living donor liver transplantation, it may also be applicable to cases with overlapping infections [23], where rehabilitation treatment has not yet been established, regardless of ICU admission or discharge. Furthermore, it could support scientific evidence for proactive mobilization [24], which is recommended for ICU-AW.

The limitations of this study include the fact that the patient's ADL did not recover to the level observed at the time of admission despite rehabilitation treatment. Furthermore, the patient was transferred to another facility for the purpose of continuing rehabilitation treatment, making it impossible to observe their progress and prognosis. Additionally, the implementation criteria for exercise therapy were based on simple indicators such as heart rate and the Borg scale, lacking objective evidence such as muscle activity measurements, which is another limitation of this study.

## Conclusions

In conclusion, this patient underwent long-term intensive care management due to postoperative complications of septic shock and bile peritonitis due to incomplete suturing of the biliary-enteric anastomosis after LDLT. We implemented rehabilitation treatment based on objective indicators and clarified outcomes, such as gradual improvement in ADL. As there are no clear guidelines for rehabilitation therapy after LDLT, especially for patients with postoperative complications, this case report may contribute to the development of rehabilitation treatment. This case was presented with oral consent from the patient.
